# Comparison of Ultra-Mini Percutaneous Nephrolithotomy and Retrograde Intrarenal Surgery for Renal Stones: A Systematic Review and Meta-Analysis from the KSER Update Series

**DOI:** 10.3390/jcm11061529

**Published:** 2022-03-10

**Authors:** Hae Do Jung, Doo Yong Chung, Do Kyung Kim, Min Ho Lee, Sin Woo Lee, Sunghyun Paick, Seung Hyun Jeon, Joo Yong Lee, on behalf of the Korean Society of Endourology and Robotics (KSER) Research Committee

**Affiliations:** 1 Department of Urology, Wonkwang University Sanbon Hospital, Wonkwang University School of Medicine, Gunpo 15865, Korea; haedojung79@wku.ac.kr; 2 Department of Urology, Inha University School of Medicine, Incheon 22212, Korea; dychung@inha.ac.kr; 3 Department of Urology, Soonchunhyang University Hospital, Soonchunhyang University College of Medicine, Seoul 04401, Korea; dokyung@schmc.ac.kr; 4 Department of Urology, Gyeongsang National University Changwon Hospital, Gyeongsang National University School of Medicine, Changwon 51472, Korea; minogi-nim@hanmail.net; 5 Department of Urology, Eulji General Hospital, Eulji University School of Medicine, Seoul 01830, Korea; icinoo0923@naver.com; 6 Department of Urology, Konkuk University School of Medicine, Seoul 05030, Korea; 20030010@kuh.ac.kr; 7 Department of Urology, Kyung Hee University Medical Center, Kyung Hee University School of Medicine, Seoul 02447, Korea; 8 Department of Urology, Severance Hospital, Urological Science Institute, Yonsei University College of Medicine, Seoul 03722, Korea; 9 Center of Evidence Based Medicine, Institute of Convergence Science, Yonsei University, Seoul 03722, Korea

**Keywords:** percutaneous nephrolithotomy, ultra-mini, retrograde intrarenal surgery

## Abstract

Miniaturized percutaneous nephrolithotomy (PCNL) and retrograde intrarenal surgery (RIRS) for renal stones have been developed to overcome the invasive disadvantages of PCNL. We aimed to compare the therapeutic effect and safety of ultra-mini percutaneous nephrolithotomy (UMPCNL) and RIRS for renal stones using an updated systematic review and meta-analysis. We searched clinical trials comparing UMPCNL and RIRS for renal stones using the PubMed, EMBASE, Cochrane Library, and Google Scholar databases up to October 2021. Seven studies were included in the current study. The renal stone size was 10–20 mm in three studies, 10–25 mm in one study, 10–35 mm in two studies, and not specified in one study. The stone-free rate of UMPCNL was higher than that of RIRS (*p* = 0.02; odds ratio (OR) = 2.01; 95% confidence interval (CI) = 1.12, 3.61). The complication rate showed no significant difference between UMPCNL and RIRS (*p* = 0.48; OR = 1.20; 95% CI = 0.73, 1.98). Regarding the operative time, UMPCNL was shorter than RIRS (*p* = 0.005; weighted mean difference (WMD) = −15.63; 95% CI = −26.60, −4.67). The hospital stay of UMPCNL was longer than that of RIRS (*p* = 0.0004; WMD = 1.48; 95% CI = 0.66, 2.31). UMPCNL showed higher efficacy than RIRS and similar safety to RIRS. UMPCNL may be a useful therapeutic option for moderate-sized renal stones.

## 1. Introduction

Urolithiasis is becoming more common across the world. In the United States, the overall prevalence of renal stones increased from 3.2% in 1980 to 10.1% in 2016 [1]. From 2000 to 2010, the hospital incidence of upper tract stones in the UK increased by 63% [2]. In Asia, the prevalence of urolithiasis has increased in recent decades (China and Japan: from 4% to 6.4% and from 4.3% to 9.0%, respectively) [3]. There has also been an increased prevalence of urolithiasis in South Korea (from 3.5% to 11.5%) [4,5]. As a result, choosing the appropriate treatment for urolithiasis is critical for enhancing the quality of life and economic aspects of stone formers.

Shock wave lithotripsy (SWL), retrograde intrarenal surgery (RIRS), and percutaneous nephrolithotomy (PCNL) are performed to treat renal stones. Open, laparoscopic, and robotic surgeries are conducted only in selected patients (i.e., those with partial and complete staghorn stones that multiple endourological approaches failed to resolve or percutaneous procedures were unsuccessful in treating) [6].

In the European Association of Urology (EAU) Guidelines on Urolithiasis, PCNL is a treatment of choice (TOC) for renal stones > 20 mm, SWL or RIRS is the first-line therapy for renal stones < 10 mm, and SWL or endourologic treatment (all PCNL and ureteroscopic interventions) can be performed for stones 10–20 mm [6]. Endourologic treatment is a TOC for lower pole renal stones of 10–20 mm, if SWL is associated with an unfavorable factor; if SWL is favorable, SWL or endourologic treatment can be performed [7]. In <20 mm renal stones, a broad expression such as “endourologic treatment” indicates that it is unclear whether PCNL or RIRS is the preferable treatment.

Recently, the percutaneous access sheath size has been minimized from the standard 30 F access to mini-PCNL (MPCNL), ultra-mini PCNL (UMPCNL), and micro-PCNL [8]. Jackman et al. first designed an MPCNL device for children in 1998 [9], and Lahme et al. first conducted MPCNL for adults in 2001 [10]. Desai et al. first performed UMPCNL (11–13 Fr metal sheath) [11]. Thus, miniaturized (mini or ultra-mini) PCNL has been developed to overcome the invasive disadvantages of PCNL; however, evidence comparing efficacy and safety is lacking. Thus, this study aimed to compare the therapeutic effect and safety of UMPCNL and RIRS for renal stones using an updated systematic review and meta-analysis.

## 2. Methods

### 2.1. Inclusion and Exclusion Criteria

The inclusion criteria of this study were as follows: (a) patients with renal stones, (b) comparison of UMPCNL and RIRS to treat renal stones, and (c) outcome measures including the stone-free rate (SFR), complications, operative time, and hospital stay. The exclusion criteria of this study were as follows: (a) studies that were not available as full texts, (b) studies without an appropriate control group, and (c) duplicated studies. This report was prepared in compliance with the Preferred Reporting Items for Systematic Reviews and Meta-Analyses statement (Supplement Table S1) [12]. This systematic review was exempt from consideration by the ethics committee or institutional review board because systematic reviews and meta-analyses do not require prior approval.

### 2.2. Search Strategy

A systematic review was performed to identify relevant articles that compared treatment for renal stones using the four English language databases PubMed, EMBASE, the Cochrane Central Register of Controlled Trials (Central), and Google Scholar up to October 2021. Search strategies were established to include Medical Subject Headings keywords such as “ultra-mini percutaneous nephrolithotomy”, “ultra-mini PCNL”, “retrograde intrarenal surgery”, “RIRS”, and combinations of these search terms.

### 2.3. Study Selection and Data Extraction

Two researchers (HDJ and DYC) screened the titles and abstracts of articles that were independently identified by the search strategy to exclude irrelevant studies. They also assessed the full text of the articles for relevance. The most relevant articles were extracted from each study, and information such as author, year of publication, country, study design, patient characteristics, and treatments was recorded, as well as outcome variables such as “SFR”, “complication rate”, “operative time”, and “hospital stay”.

### 2.4. Study Quality Assessment

We used the Cochrane Risk of Bias (ROB) tool for randomized control trials (RCTs), and the methodological index for non-randomized studies (MINORS).

Grading of the quality of evidence was performed by our researchers independently (HDJ and DKK) using the Scottish Intercollegiate Guidelines Network (SIGN) checklist, which comprises various study types, including systematic reviews and meta-analyses, RCTs, cohort studies, case–control studies, diagnostic studies, and economic studies. All disagreements regarding the quality assessment results were resolved after discussion with a third reviewer (JYL).

### 2.5. Statistical Analysis

The odds ratios (ORs) and 95% confidence intervals (CIs) were calculated and reported for dichotomous variables. The weighted mean difference (WMD) and 95% CI were calculated for the continuous variables. The chi-squared test, with *p* values less than 0.05, was used to evaluate statistical heterogeneity, and the *I*^2^ statistic was used to quantify heterogeneity [13]. If the reported *I*^2^ statistic was less than 50%, we applied the fixed-effects model; otherwise, the random-effects model was used. The Higgins *I*^2^ statistic was calculated as follows:

$$I^{2}=\frac{Q-df}{Q}\times 100\%$$

where “*Q*” is the Cochrane heterogeneity statistic, and “*df*” is the degrees of freedom. All meta-analyses were performed using Review Manager, version 5.4.1 (RevMan, Copenhagen, Denmark: The Nordic Cochrane Center, The Cochrane Collaboration, Oxford, UK, 2020).

Subgroup analysis was performed in two patient groups according to the renal stone location. If the patient had lower pole renal stones, we classified them as “lower pole”. Patients were considered “not specified” if the renal stone location could not be determined. This systematic review is registered in PROSPERO, CRD42021283309.

## 3. Results

### 3.1. Eligible Studies

**Figure 1:**
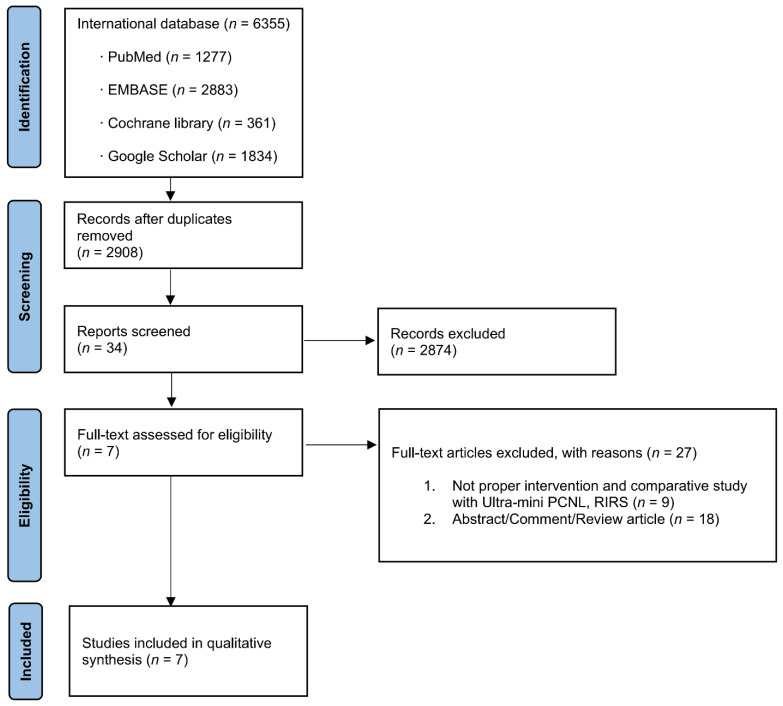
Study flow chart.

A total of 2908 studies were identified for potential inclusion. After a full-text review, seven articles were identified as relevant to the current study and selected for inclusion in the meta-analysis (Figure 1) [14,15,16,17,18,19,20].

### 3.2. Characteristics of the Included Studies

**Figure 2:**
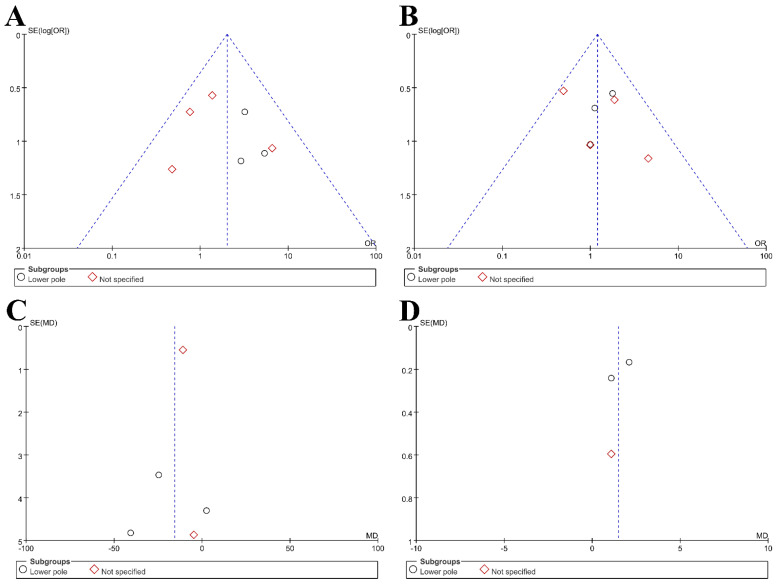
Funnel plots: (**A**) stone-free rate (SFR); (**B**) complication rate; (**C**) operative time; (**D**) hospital stay.

**Figure 3:**
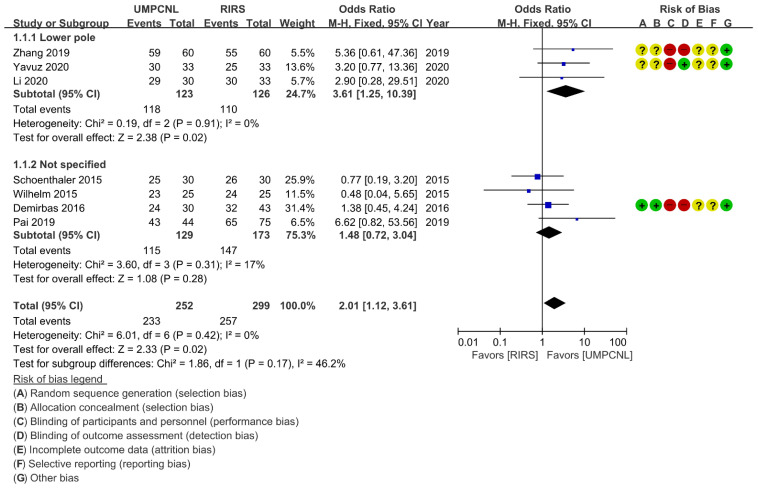
Comparison of ultra-mini percutaneous nephrolithotomy (UMPCNL) and retrograde intrarenal surgery (RIRS) in terms of the SFR.

**Figure 4:**
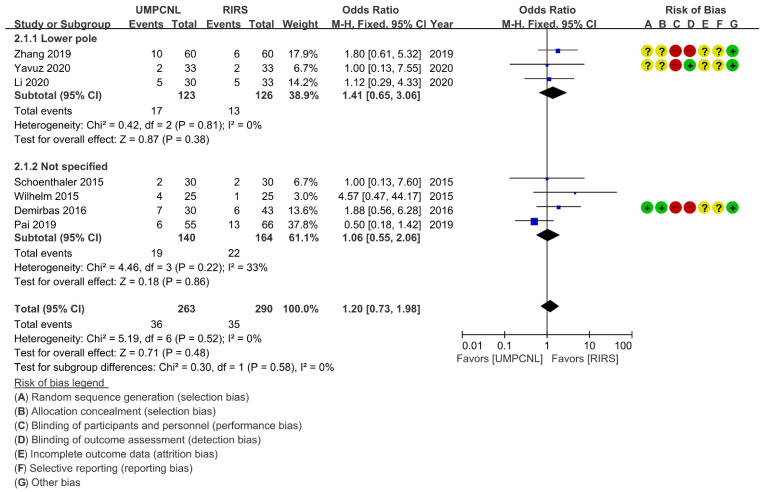
Comparison of UMPCNL and RIRS in terms of the complication rate.

**Figure 5:**
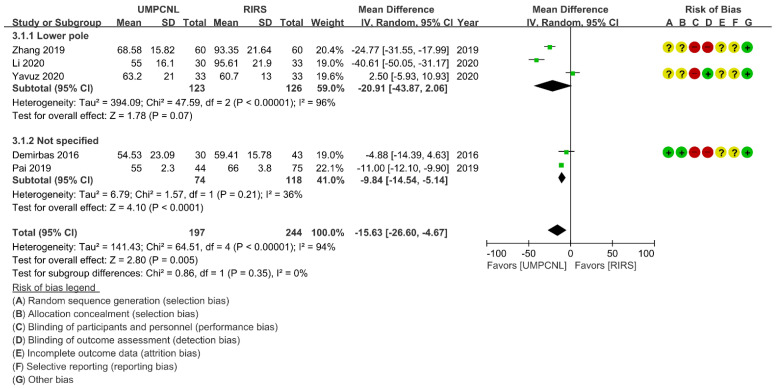
Comparison of UMPCNL and RIRS in terms of the operative time.

**Figure 6:**
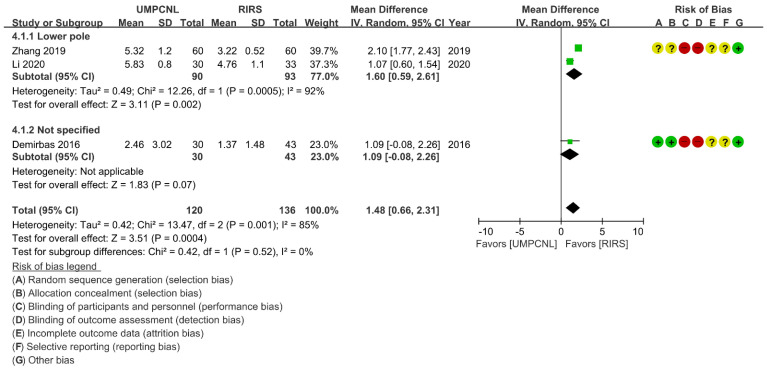
Comparison of UMPCNL and RIRS in terms of the hospital stay.

The characteristics of the seven included studies are shown in Table 1 [14,15,16,17,18,19,20]. These comparative studies described patients who had undergone UMPCNL and RIRS for renal stones. The included studies were published between January 2015 and May 2020. Five studies were performed in Europe (two studies from Turkey, one study from the UK, one study from Germany, and one study from Germany and the UK) [14,16,17,18,19]. Two studies were performed in China [15,20]. Three studies were selected based on the inclusion of a lower pole group [15,19,20]; four other studies were chosen but did not specify the patient group [14,16,17,18]. The results of the quality assessment of the included studies are shown in Table 1 and were found to be acceptable. Two studies were rated as 1+, one study was rated as 1−, and four studies were rated as 2+. Funnel plots of the meta-analyses are shown in Figure 2. There was little evidence of publication bias in most of the included studies. The ROB for RCTs is displayed in Figure 3, Figure 4, Figure 5 and Figure 6. The MINORS scores for non-RCTs are displayed in Table 2. All studies were reasonable.

### 3.3. Heterogeneity Assessment

Regarding the SFR and complication rate, few heterogeneities were found (*p* = 0.42; *I*^2^ = 0%, and *p* = 0.52; *I*^2^ = 0%, respectively). Thus, fixed-effects models were used to compare the SFR and the complication rate between UMPCNL and RIRS (Figure 3). Regarding the operative time and hospital stay, heterogeneities were found (*p* < 0.01; *I*^2^ = 94%, and *p* < 0.01; *I*^2^ = 85%, respectively). Thus, random-effects models were used to compare the operative time and hospital stay between UMPCNL and RIRS (Figure 4, Figure 5 and Figure 6).

### 3.4. Stone-Free Rate

The SFR was compared between UMPCNL and RIRS in seven studies [14,15,16,17,18,19,20]. The definition of SFR in the included studies revealed some variation in imaging and follow-up time for identifying SFR; thus, we assessed it as the SFR at the final follow-up (Table 1). UMPCNL showed a significantly higher SFR than RIRS (*p* = 0.02; OR = 2.01; 95% CI = 1.12, 3.61; *I*^2^ = 0%) (Figure 3). Three studies involved lower pole groups [15,19,20], whereas four studies did not specify the patient group [14,16,17,18]. Although the subgroup differences indicated no statistically significant subgroup effect (*p* = 0.17), higher SFRs were found in the lower pole groups treated with UMPCNL than those treated with RIRS, as the subgroup analysis data revealed (*p* = 0.02; OR = 3.61; 95% CI = 1.25, 10.39; *I*^2^ = 0%). There were no significant differences between UMPCNL and RIRS in the not specified groups (*p* = 0.28; OR = 1.48; 95% CI = 0.72–3.04; *I*^2^ = 17%).

### 3.5. Complication Rate

The complication rate, according to the Clavien–Dindo classification, was compared between UMPCNL and RIRS in seven studies [14,15,16,17,18,19,20]. The complication rate showed no significant difference between UMPCNL and RIRS (*p* = 0.48; OR = 1.20; 95% CI = 0.73, 1.98; *I*^2^ = 0%) (Figure 4). Three studies involved lower pole groups [15,19,20], whereas four studies did not specify the patient group [14,16,17,18]. The results of subgroup analysis revealed no significant differences between UMPCNL and RIRS in the lower pole and not specified groups (*p* = 0.38; OR = 1.41; 95% CI = 0.65, 3.06; *I*^2^ = 0%, and *p* = 0.86; OR = 1.06; 95% CI = 0.55, 2.06; *I*^2^ = 33%, respectively).

### 3.6. Operative Time

The operative time was compared between UMPCNL and RIRS in five studies [14,15,16,19,20]. The operative time of UMPCNL was significantly shorter than that of RIRS (*p* = 0.005; WMD = −15.63; 95% CI = −26.60, −4.67; *I*^2^ = 94%) (Figure 5). Three studies involved lower pole groups [15,19,20], whereas two studies did not specify the patient group [14,16]. Although the subgroup differences indicated no statistically significant subgroup effect (*p* = 0.35), the subgroup analysis results revealed a shorter operative time in the not specified groups treated with UMPCNL compared to that in the not specified groups treated with RIRS (*p* < 0.0001; WMD = −9.84; 95% CI = −14.54, −5.14; *I*^2^ = 36%), but no significant differences were found between UMPCNL and RIRS in the lower pole groups (*p* = 0.07; WMD = −20.91; 95% CI = −43.87, 2.06; *I*^2^ = 96%).

### 3.7. Hospital Stay

The hospital stay was compared between UMPCNL and RIRS in three studies [14,15,20]. The hospital stay of UMPCNL was significantly longer than that of RIRS (*p* = 0.0004; WMD = 1.48; 95% CI = 0.66, 2.31; *I*^2^ = 85%) (Figure 6). Two studies involved lower pole groups [15,20], whereas one study did not specify the patient group [14]. Although the subgroup differences indicated no statistically significant subgroup effect (*p* = 0.52), a longer hospital stay in the lower pole groups treated with UMPCNL than those treated with RIRS was revealed in the subgroup analysis results (*p* = 0.002; WMD = 1.60; 95% CI = 0.59, 2.61; *I*^2^ = 92%). No significant differences were found between UMPCNL and RIRS in the not specified groups (*p* = 0.07; WMD = 1.09; 95% CI = −0.08, 2.26; *I*^2^ = not applicable).

## 4. Discussion

A systematic review of nearly 12,000 patients revealed the following incidence rates of PCNL-related complications: fever, 10.8%; transfusion, 7%; thoracic complication, 1.5%; sepsis, 0.5%; organ injury, 0.4%; embolism, 0.4%; urinoma, 0.2%; death, 0.05% [21]. Tract size is related to post-PCNL bleeding due to injury to the surrounding structure resulting from multiple incremental dilatations [22]; thus, miniaturized PCNL was developed for smaller tract sizes. The access sheath size can be used to categorize miniaturized PCNL (i.e., MPCNL, UMPCNL, and micro-PCNL have sizes of 14–20 Fr, 11–13 Fr, and 4.85 F, respectively) [23]. Super-mini-PCNL (SMP) is a new miniaturized PCNL that consists of an 8 Fr super-mini nephroscope with a 12 or 14 Fr irrigation–suction sheath for increased irrigation and stone clearance [24]. The miniaturization of equipment can reduce invasiveness, and numerous articles concerning miniaturized PCNL showed an excellent SFR [25,26]. Transfusion rates increased with increasing sheath size (18 F and below: 1.1%; 24 F and 26 F: 4.8%; 27 F, 28 F, and 30 F: 5.9%; 32 F, 33 F, and 34 F: 12.1%) [22].

The EAU Urolithiasis Guidelines Panel conducted a systematic review assessing the efficacy and safety of miniaturized PCNL (<22 F) to remove renal calculi [27]. In miniaturized and standard PCNL, the SFRs were comparable. Smaller instruments are associated with significantly lower blood loss, but the operation time is significantly longer. No significant differences were found in other complications in that study. It was concluded that miniaturized PCNL is at least as effective and safe as standard PCNL. However, the study described the necessity for more well-designed RCTs because the quality of evidence of the studies included in its meta-analysis was poor, with only two RCTs. Most of the studies were single-arm case series and non-randomized comparative studies. Additionally, the tract sizes and stone types were heterogeneous. Therefore, a high risk of bias and confounding were observed.

UMPCNL comprises a 3.5 Fr telescope, a 6 Fr inner sheath, and an 11 Fr or a 13 Fr outer sheath with an irrigation port [28]. Desai et al. published the first article on the initial experience with UMPCNL for <2 cm kidney stones in 2013 [11]. In their study, the mean operative time was 59.8 ± 15.9 (30–90) min. The SFR was 97.2% one month after UMPCNL, and the average hospital stay was 3.0 ± 0.9 (2–5) days. The complication rate was 16.7% (6/36) and complications included sepsis (5.6% (2/36)), urinary extravasation (2.8% (1/36)), and fever (8.3% (3/36)). No patient required a blood transfusion. However, to date, the acceptable criterion for using miniaturized PCNL has been renal stones < 3.0–3.5 cm; UMPCNL is thought to be suitable for stones < 1.5 cm [11,29,30,31]. Recently, Haghighi et al. conducted a randomized clinical trial comparing UMPCNL and standard PCNL for renal or upper ureteric stones of 10–20 mm [32]. UMPCNL showed less blood loss, a shorter hospital stay, and less postoperative pain than standard PCNL. Additionally, no differences were found in the operative time and SFR.

However, RIRS was developed to replace invasive PCNL. In 1964, Marshall described his first experience with fiber optics in urology [33]. Bagley et al. reported the first clinical results of a flexible ureteroscope [34]. After that, the introduction of actively deflectable ureteroscopes, irrigation channels, the smaller diameter of the flexible ureteroscope with stronger durability, and the digital flexible ureteroscope, as well as the development of laser lithotripsy, led to the popularity of RIRS in the treatment of nephrolithiasis. However, RIRS for large renal stones > 20 mm may require multiple RIRS sessions [35,36]. Therefore, PCNL is recommended as the first treatment for renal stones > 20 mm [37]. RIRS for lower pole renal stones > 10 mm has a risk of failure in the narrow and acutely angled lower pole infundibulum, even with maximal deflection [37].

Gao et al. published a meta-analysis for miniaturized PCNL and RIRS [38]. In their meta-analysis, only two studies were analyzed to compare UMPCNL and RIRS [14,17]. The SFR and operative time of UMPCNL were similar to those of RIRS. The complication rate of UMPCNL was higher than that of RIRS, without a significant difference. Additionally, the hospital stay for UMPCNL was longer than that for RIRS, but the difference was not significant.

However, in our meta-analysis, the SFR of UMPCNL was higher than that of RIRS (*p* = 0.02; OR = 2.01; 95% CI = 1.12, 3.61), and the operative time was shorter for UMPCNL than for RIRS (*p* = 0.005; WMD = −15.63; 95% CI = −26.60, −4.67). The complication rate showed no significant difference between UMPCNL and RIRS regardless of the renal stone location (*p* = 0.48; OR = 1.20; 95% CI = 0.73, 1.98). Additionally, postoperative pain was similar between UMPCNL and RIRS or showed a higher visual analog scale (VAS) pain score in UMPCNL than in RIRS [14,18]. UMPCNL, on the other hand, had a lower postoperative VAS pain score than PCNL, according to Haghighi et al. [32]. The hospital stay of UMPCNL was significantly longer than that of RIRS (*p* = 0.0004; WMD = 1.48; 95% CI = 0.66, 2.31). Even though the hospital stay of UMPCNL was longer than that of RIRS, the overall cost was lower in UMPCNL than in RIRS [17,19,20].

Our study has limitations. First, there was some discrepancy in the renal stone size of the analyzed studies. The stone size classification is crucial because it is related to the SFR. Second, there were some inconsistencies in the definition of the SFR among the studies included. The SFR could be affected by the variation in follow-up imaging and timing [39,40]. Third, the difficulty in the accessibility of the flexible ureteroscope in the lower pole anatomy was not considered in the analyzed studies; this condition is one of the drawbacks of flexible ureteroscopic surgeries. However, an improved flexible ureteroscope with a smaller diameter and a larger deflection angle provides an opportunity for further analysis. Fourth, the number of analyzed studies was small. Because UMPCNL is not popular, few comparative studies are available. Although our study has some limitations, UMPCNL showed better surgical results than RIRS in our meta-analysis. However, prospective randomized trials with large sample sizes are needed.

## 5. Conclusions

UMPCNL showed higher efficacy than RIRS and similar safety compared to RIRS. The overall cost was lower in UMPCNL than in RIRS. Even though UMPCNL had less postoperative pain than PCNL, patients may experience more pain with UMPCNL than with RIRS. As a result, after the preoperative counseling of patients, UMPCNL can be recommended for moderate-sized renal stones.

## Figures and Tables

**Table 1:** Characteristics of the included studies.

Author Year	Country	Design	Procedure (Access Sheath Size)	Inclusion Criteria	No. of Patients	Mean Age	Body Mass Index	Definition of Stone-Free	Complication		Quality Assessment(SIGN)
									Clavien I–II	Clavien III–IV	
Schoenthaler et al., 2015 [17]	Germany/UK	Retrospective	Ultra-mini PCNL (13Fr)	Renal stones 10–20 mm	30	54.3 (19–72)	29.9 (18.7–42.1)	Intra-operative inspection/fluoroscopy and postoperative sonography		21	2+
			RIRS		30	56.3 (18–76)	28.7 (19.1–38.3)			21	
Wilhelm et al., 2015 [18]	Germany	Prospective	Ultra-mini PCNL (13Fr)	Renal stones 10–35 mm	25	51.6 15–75)	29.5 (18.8–43.0)	≤3 mm as assessed by endoscopic inspection and immediate postoperative ultrasound, or low-dose CT after 4–8 weeks	4 (Clavien II or III)		2+
			RIRS		25	51.4 (19–77)	28.4 (18.4–38.6)		1 (Clavien II or III)		
Demirbas et al., 2016 [14]	Turkey	RCT	Ultra-mini PCNL (14Fr)	Renal stones 10–25 mm	30	43.7 ± 14.6		Complete removal or ≤3 mm in low-dose non-contrast CT after one month	2	5	1+
			RIRS		43	48.7 ± 16.8			3	3	
Zhang et al., 2019 [20]	China	RCT	Ultra-mini PCNL (13Fr)	Lower pole renal stones 10–20 mm	72	48.9 ± 11.1	24.3 ± 3.0	Complete removal or ≤3 mm examined by CT	7	3	1−
			RIRS			50.1 ± 11.9	24.3 ± 3.1		5	1	
			SWL		66	50.5 ± 12.6	24.3 ± 3.1		3	1	
Pai et al., 2019 [16]	UK	Retrospective	Ultra-mini PCNL (13Fr)	Renal stones	44	54	32.6	Three months postoperatively with either plain radiography or renal ultrasonography	6		2+
			RIRS		75	57	29.6		13		
Yavuz et al., 2020 [19]	Turkey	RCT	Ultra-mini PCNL (11/12Fr)	Lower pole renal stones 10–20 mm	33	45.2 ± 12.7	24.5 ± 2.6	Complete removal or ≤3 mm in low-dose non-contrast CT after 3 months	2 (≥Clavien II)		1+
			RIRS		33	48.1 ± 13.1	25.4 ± 2.8		2 (≥Clavien II)		
			Micro-PCNL (4.8Fr)		35	42.8 ± 13.5	25.1 ± 3.0		3 (≥Clavien II)		
			Mini PCNL (15/16Fr)		34	42.3 ± 12.7	24.6 ± 3.7		3 (≥Clavien II)		
			Standard PCNL (23/24Fr)		33	49.2 ± 10.9	25.9 ± 2.9		2 (≥Clavien II)		
Li et al., 2020 [15]	China	Retrospective	Ultra-mini PCNL (9.5/11.5Fr)	Lower pole renal stones 15–35 mm	30	52.5 ± 11.2 (22–70)	23.5 ± 3.0 (16.4–27.1)	One-month SFR and three-month SFR with ≤2 mm in plain abdominal radiography	5		2+
			RIRS		33	49.1 ± 11.5 (26–77)	24.2 ± 3.0 (19.1–30.4)		5		

PCNL, percutaneous nephrolithotomy; RIRS, retrograde intrarenal surgery; RCT, randomized controlled trial; SWL, shock wave lithotripsy; CT, computed tomography. The quality assessment was performed using the Scottish Intercollegiate Guidelines Network (SIGN) checklist. 1+ means well-conducted RCTs with a low risk of bias. 1− means RCTs with a high risk of bias. 2+ means well-conducted cohort studies with a low risk of bias. 2− means cohort studies with a high risk of bias.

**Table 2:** MINORS score in non-randomized studies included in the review.

	Schoenthaler et al., 2015 [17]	Wilhelm et al., 2015 [18]	Pai et al., 2019 [16]	Li et al., 2020 [15]
A clearly stated aim	2	2	2	2
Inclusion of consecutive samples	2	2	2	2
Prospective collection of data	0	2	0	0
Endpoints appropriate for the aim of the study	2	2	2	2
Unbiased assessment of the study endpoint	0	0	0	0
Follow-up period appropriate for the aim of the study	2	2	2	2
Loss to follow-up less than 5%	2	2	2	2
Prospective calculation of the study size	0	0	0	0
An adequate control group	2	2	2	2
Contemporary groups	2	2	2	2
Baseline equivalence of groups	2	2	2	2
Adequate statistical analyses	2	2	2	2
Total	18	20	18	18

MINORS, methodological index for non-randomized studies. The items are scored 0 (not reported), 1 (reported but inadequate), or 2 (reported and adequate). The global ideal score is 16 for non-comparative studies and 24 for comparative studies.

## Data Availability

Data are contained within the article or Supplementary Materials.

## Supplementary Materials

The following supporting information can be downloaded at: https://www.mdpi.com/article/10.3390/jcm11061529/s1, Table S1: PRISMA Checklist.
